# The Jenks Prize Essay

**Published:** 1889-06

**Authors:** 


### *THE JENKS PRIZE ESSAY*[1].

DR. STRAHAN'S essay, to which was accorded the "Jenks prize" of the College of Physicians of Philadelphia, and for which the author is complimented by the awarding committee "upon its remarkable excellence," deserves special notice, both for the prominence it has thereby received, and on account of the widespread interest and discussion the subject with which it deals is attracting. The essay is lengthy, and to review it in detail would be a matter of no little difficulty, had not a previous editorial review in this JOURNAL considered at length a book upon which Dr. Strahan draws most extensively for nearly all the most important portions of his production. This applies not only to the subject-matter entirely

1. The Diagnosis and Treatment of Extra-uterine Pregnancy. By John Strahan, M. D., M. Ch., M. A. O. (Royal University of Ireland); Fothergillian gold medalist of the London Medical Society, 1886, for essay on Typhoid Fever; Member of the Ulster Medical Society, and British Medical Association, etc., etc.; Jenks Prize Essay of the College of Physicians of Philadelphia. Philadelphia: P. Blakiston, Son & Co. 1889.

Mr. Tait's own, but also to many quotations introduced by him to fortify and illustrate the positions assumed in his lectures. Dr. Strahan has, nevertheless, introduced many new references into his essay, and with some of these it will be necessary to deal, especially on the subject of diagnosis in reference to the electrical treatment. The mechanism of the production of ectopic gestation, as explained by Mr. Tait, and proven by the researches of Hart and Carter, Dr. Strahan accepts without question, giving Tait's "genealogical" table in full.

So far as ovarian pregnancy is concerned, Dr. Strahan speaks too dogmatically when he affirms that Mr. Tait has disposed satisfactorily of Spiegelberg's reported case of ovarian pregnancy. Mr. Tait does not believe that this form of pregnancy is probable. He does not deny its possibility, and frankly acknowledges "that the emine nce of the observer, and the manifest care with which all his records are given, make it quite possible that his conclusions are correct." (Lectures, p. 11.)

As to the occurrence of primary intra-peritoneal pregnancy, Dr. Strahan expresses no opinion of his own, though he tacitly accepts Tait's, fortified as it is by the researches of Hart and Carter. Indeed, so far in the essay both the subject-matter and the ideas are almost reproductions of Mr. Tait's own expression.

Under the caption, Diagnosis of Extra-uterine Pregnancy previous to Rupture, Dr. Strahan discusses perhaps the most vexed portion of his whole subject. His conclusions, we believe, are in the main correct, though many data at hand to fortify his opinions are passed over without notice, while some suggestions almost sufficiently fantastical for a Venetian carnival are considered with a gravity they in no wise merit. Such, for instance, is Rasch's assertion of the diagnosis of uterine pregnancy as early as the seventh week, by fluctuation in some part of the uterus.

The question of diagnosis of tubal pregnancy by the fetal heart sounds, it is evident, depends upon the period at which rupture occurs, or whether the fetus has survived primary rupture. Bernutz and Goupil, (Vol. I., p. 253, *Syd. Soc. Trans.*,) without reckoning extreme cases, in which rupture occurred at from one to eight months, place the period at which this accident occurs between the second and fourth month. Mr. Tait places the period between the twelfth and fourteenth week. The weight of authority places the positive hearing of the fetal heart beyond both of these latter periods. So that diagnosis of ectopic gestation, by auscultation of the fetal heart, can be dismissed, except, perhaps, as the rarest of curiosities, not valuable, but perplexing. The affirmation that ectopic gestation is more easily diagnosticated in the early months than is normal pregnancy, whether it be affirmed by philosopher or fool, is, in either case, arrant nonsense. They who have had the most experience will contradict such assertion most emphatically. When probabilities point in a certain direction, the imagination is apt to furnish facts to strengthen such probabilities. Even if, as Routh affirms, he has heard the uterine souffle before the third month, such sign is worthless, because it does not differentiate normal from misplaced pregnancy. As a rule, those who are the most positive in their ability to diagnosticate this condition have had the least practical experience in it. So far as Rasch's diagnosis by fluctuation is concerned, we do not believe it worthy of any credence whatever. Dr. Rasch's tactile sense must have been almost microscopical, putting Pope to shame when he writes:

> "Why has not man a microscopic eye?
> For this same reason: man is not a fly."

Dr. Strahan's acceptance of Rasch's "new (?) sign" of pregnancy,—increased desire of urination, especially at night,—"not noticed in the text-books," is naive, though not critical, especially as it is found in all obstetrical books as one of the physical signs of this condition. His suggestion, also, that in the absence of the fluctuation symptom, the sound could be used, is dangerous advice and dangerous gynecology. In suspected pregnancy, until it is definitely settled that the uterus is empty, the sound is the last instrument to be thought of. Other signs of pregnancy noticed may be passed over as adding nothing positive to the possibilities of diagnosis.

The diagnosis of intra-uterine from extra-uterine pregnancy is the next phase of the question discussed. Here, again, hinges much of the claim for positive diagnosis. If it is granted that pregnancy is present, and that it is not intra-uterine, of course it must be ectopic. This is almost the logic of the positivist.

If from the date of a supposed conception, or sexual congress, we could have a woman constantly under observation, there is but little doubt that pregnancy, if it occurred, could be diagnosticated, and if abnormal, so decided. This status of affairs, then, is for the most part impossible, and can be dismissed. The next proposition of the positivists necessary to establish is, that the symptoms of ectopic gestation are pronounced, regular, and practically pathognomonic, previous to the period of rupture,—say to the fourteenth or fifteenth week. In addition, it must be the rule that the patient, by reason of peculiar symptoms, has her own suspicions excited that some abnormal process is going on in her economy, and resorts to the physician for advice or relief. Here, as Dr. Strahan justly points out, we must not confound those cases in which primary rupture has been survived, and diagnosis has been made either at full term or after missed labor, or by ausculting the fetal heart sounds after the fetus has become viable; neither are we to permit the evidence of such cases to mislead us into supposing that the history of all cases is uniform in all particulars. It is obvious that if there is nothing to excite the woman's suspicion previous to rupture, the surgeon will have no means, except accidentally, to investigate any concurrent phenomena that may attend the condition. Now, unfortunately for absolute diagnosis, the symptomatology in the greater number of cases is not uniform, neither physically nor rationally. Mr. Tait affirms that in none of his cases operated on after rupture had the condition been previously diagnosticated, and that a great majority of his patients had no suspicion that they were pregnant. W. Japp. Sinclair (*British Medical Journal*, Jan. 21, 1888, p. 127,) adds his testimony of two cases with rupture, in neither of which was pregnancy suspected. He says:

"There was an entire absence of symptoms pointing to abnormal pregnancy in both cases. Neither of them could exceed two months, and in both the Fallopian tube was distended to the bursting point without giving rise to such uneasiness as to evoke any complaint from the patient."

Can anything be stronger than this testimony as to the unreliability of *unusual* symptoms in ectopic pregnancy? Mr. Tait's case, in whom the accident occurred twice, the second time with fatal result, with no symptoms to cause discomfort or apprehension, is well known. McNaught (*British Medical Journal*, ibid ante, p. 129,) reports a case in which rupture occurred at the second month, the woman suspecting herself pregnant, with nothing unusual to attract her attention. Another case lately under our notice, not reported, had a history of one missed period, a fall five weeks before, severe pain, and no previous inaptitude for concep tion. After the fall, the woman had passed what was probably decidua, but up to that time did not think herself pregnant, and, afterward, thought she had miscarried. Than this, could anything be more misleading? Dr. Strahan's comment on this sort of cases is at once sensible and pithy: "If we could arrange the circumstances, we could, no doubt, often diagnose either extra- or intra-uterine pregnancy." The trouble is, the symptoms arrange themselves, rarely orderly—never uniformly—and before we can dispose them into a logical sequence, rupture occurs, when the diagnosis makes itself, and the surgeon takes the credit. Now, while subjective symptoms may be absent, so far as anything unusual is concerned, the same may be true of the physical signs. Menstruation during the first months may be regular, or so slightly irregular as to excite no suspicion; or, again, it may be so profuse as to suggest miscarriage; or, yet again, it may be absent without other signs of pregnancy. As to the diagnostic value of the decidua—this, too, is variable. If it is shed, as in the case cited above, it is often interpreted as marking a miscarriage. Again, it may be retained until after rupture, (*Lon. Ob. Trans.*, Vol. I., p. 101,) when it is clearly of no value whatever. Then, again, there is reliable evidence that the decidua may be absent. (Graily Hewitt, *Lon. Ob. Trans.*, p. 49, Vol. II.; communicated for Grace.) In this case, the woman did not believe she was pregnant, though she had missed a period two weeks. Rupture occurred without hemorrhage from the vagina. *Post mortem* revealed rupture of a cyst the size of a walnut, in the left tube. The cyst contained what resembled a decidual lining. " The uterus was not at all enlarged or congested, and on laying it open no deciduous membrane was seen. It presented the usual appearance of the unimpregnated state." Though Campbell has, according to Dr. Strahan, explained the absence of the decidua, such cases as this must remain a puzzle, and defy absolute diagnosis. With such a history, "we can guess, but not affirm." The case, then, demonstrates both that in tubal pregnancy the uterus may be small, the decidua absent, and vaginal hemorrhage wanting. That this case is exceptional, is not denied. It is, nevertheless, germain to the subject in proving that histories and symptoms are misleading.

The fallacy in the argument of the positivists is that, like Routh, (*British Medical Journal*, Dec. 4, 1886, p. 1094, quoted by Strahan,) they lay down a geometrical proposition, draw and letter the figure, and claim that every case can be demonstrated by it.

Says Routh: " Four signs make the diagnosis easy in the first class of cases, viz., up to the third month: (1) the general signs of pregnancy; (2) the shifting of the uterus to one side by a growing tumor on the other; (3) stoppage of the catamenia, and often, subsequent delivery of the whole or part of a decidua; (4) detection of uterine souffle with vaginoscope, and not through abdominal walls."

Here are, as has been said, four propositions, categorically laid down as if invariable, when the truth is they may, one or all, vary, or fail, or be complicated, so that, instead of leading to the demonstration of any one condition, they reduce it, according to the law of probabilities, to the possibility of one of three or four. The case of Aveling, cited by Dr. Strahan as easy of diagnosis, (p. 20, and *British Medical Journal*, Dec. 4, 1886,) was certainly diagnosticated after some rupture had occurred, the patient having collapsed twice.

We do not agree that it should be admitted as a case of diagnosis previous to rupture. Petch's case (ibid, p. 1092,) was diagnosticated conclusively by fetal heart sounds. The whole history of the case was misleading, as Strahan insists, and it cannot be quoted as a diagnosis previous to rupture. In view of his own facts, which are weightier than Dr. Strahan has considered them, his verdict (p. 23) as to the diagnosis of extra-uterine pregnancy, up to the period of primary rupture, is just:

"Practically, the fact seems to be that, in the vast majority of the cases, no opportunity for diagnosis is given. The women have either no symptoms, or their symptoms are not such as to lead them to seek advice."

On page 25, Dr. Strahan quotes Herman, obstetric physician to the London Hospital, as follows:

"There are a great number of published cases in which the diagnosis of extra-uterine gestation has been made, galvanism has been used, and the tumors have shrunk away. But the great mass of literature of this kind is far from convincing. The papers of Thomas (*Amer. Gyn. Trans.*, Vol. VII., p. 219,) and Garrigues (ibid, p. 184,) contain many cases in which the diagnosis rests upon such slender grounds that it is impossible not to suspect error. The strength of a chain is its weakest link, and when one finds adduced, as scientific evidence, cases of an extremely doubtful kind, without, apparently, a sufficient sense of the possibility of mistake, one is apt to judge of the strength of the whole chain of evidence by these weak links."

Dr. Herman's judgment of these cases is fair, and Dr. Strahan had been wise to analyze them, with a view of discovering their inconsistencies, their disagreement in symptoms, and the scanty data which are taken as sufficient to assure the existence of extra-uterine gestation. These cases of Thomas are generally quoted, owing to his claim of positive diagnosis, as really proven; while the fact is, many of them would not stand an instant before the ordinary rules of evidence. All the first four cases reported by Thomas in the paper alluded to died. In three, puncture was made; in one, rupture occurred. In none was there a *post mortem*. Case six was not diagnosticated; case seven has defective history, and, though diagnosis is claimed, it was after death of the fetus, and soon after fetal bones were discharged. Case eight was a missed pregnancy, in which, after drawing four quarts of a sero-purulent fluid with an aspirator, the diagnosis of ectopic gestation was made by feeling a mass like a child rolling around within the abdomen. Case nine is also a missed labor, and is negative so far as early diagnosis is concerned. The same is true of case ten. Case eleven died of rupture, without diagnosis having been made, though the question of its existence was considered at a consultation. Case twelve was diagnosticated after rupture. Case thirteen seems to be a doubtful one to Dr. Thomas himself. He says: "Since this time I have lost sight of my patient, and I cannot say whether any evidences have been established settling the validity of the diagnosis."

Case fourteen was considered by Dr. Peaslee to be a rapidly-developing fibroid. The diagnosis was not made until after the second attack of very severe pain and flooding, subsequent to the third month. The diagnosis was, therefore, after rupture. In case fifteen, after the application of electricity, the fetus was expelled through the vagina. Dr. McBurney considered it a tubal, while Dr. Thomas thought it an interstitial, pregnancy. May it not have been normal? Case sixteen, at best, is uncertain, as is his seventeenth. Case eighteen, Lusk's, had not menstruated for two months. A vaginal examination revealed slight increase in the size of the uterus, *but nothing else which specially attracted his attention*. Not until the patient nearly collapsed from severe pain (probably hemorrhage) was the true nature of the case divined. Electricity—recovery.

This case certainly does not owe its success to early diagnosis, whatever may have been the effect of the electricity. In case nineteen a difference of opinion existed between Drs. Thomas and Emmet; the latter thinking it ectopic,—the former divided in his opinion between ectopic pregnancy and a normal pregnancy, complicated with a fibroid in the uterine wall. In this case the history is just as plain as in others in which Dr. Thomas seems to have had no doubt at all as to the existence of extra-uterine pregnancy. Case twenty went to the end of the third month, without remarkable symptoms. A globular mass, posterior and to the side, had been regarded as the fundus retroverted; collapse led to diagnosis. Case twenty-one, though suffering more markedly than others in which diagnosis was reached, was not decided to be ectopic until symptoms of rupture came on. After such a recital, we do believe the evidence contained is sufficient to justify a claim for positive diagnosis. The electrical treatment need not here be touched upon.

In *Am. Gyn. Trans.*, Vol. IX , Dr. Thomas reports six additional cases of extra-uterine pregnancy coming under his notice. This report, preliminary to the record of the cases, contains the following:

"Need I say how difficult such diagnosis is at a period sufficiently early to prevent rupture of a distended Fallopian tube, which usually gives way before the fourth month of gestation has far advanced?"

Again he says (*Am. Sys. Gyn.*, Vol. II., p. 188,):

"After all is said with regard to the diagnosis of ectopic gestation, it must be added that a positive conclusion is very generally difficult and often impossible."

In his report, p. 166, supra, he says of the first case:

"In other words, I felt sure the case was one in which positive diagnosis was impossible."

In case second, after the uterus had been curetted, and an attack in which the patient was reported to have fainted, extra-uterine pregnancy was suspected, but examination did not reveal any tumor. Dr. Thomas agreed in diagnosis after the second rupture. After the passage of the current, the "tumor was firmer and not increased." Here, certainly, was no early diagnosis. In case three, there is a history of suppressed menstruation, nausea, pain in breasts, and in about three months, patient unable to leave her bed. Constant pain. Seized two or three times in twenty-four hours with violent paroxysms of pain, after which she fell into complete syncope. Electricity, April 21st. September 15th, she goes out for short walks. Concluding, Dr. Thomas says: "I freely confess I am not entirely positive about the correctness of my diagnosis." He confesses this, although the mass had become less tense and less painful on pressure. Why this history should be regarded as not sufficient for positive diagnosis, if any mere history even is, is not plain.

Case four has a history scarcely as clear as that of case three, yet it is a positive diagnosis. Case five, (C. K. Briddon,) after two months' absence of menses, had vaginal flooding of clotted blood. Some time after had sudden pain and had to sit down. Second attack with collapse. Operation ; death. Here again is no early diagnosis. In case six, the woman passed all through her gestation without suspicion of anything abnormal, the condition only being discovered when labor came on, the uterus being examined at that time. It will be seen that Herman's judgment, quoted above, is upheld by the facts here recorded. Space does not permit us to follow up the discussion of Dr. Thomas's paper, but if the reader cares to refer to it, he will find such a divergence of opinion, as will satisfy him that positive diagnosis is far from being substantiated, and as a rule is almost mythical.

Dr. Strahan regards the persistence of ectopic gestation in the tube, up to full time, as impossible with the present pathology. In this the consensus of authority is by all odds in his favor, no matter what individual observers may report.

As to diagnosis after the shock of primary rupture is over, the thing most to be relied upon is the history of rupture, and the pathological fact that most of these intra-peritoneal hemorrhages are due to ectopic rupture. When Dr. Strahan says, (p. 43,) "There is no possible source of error if we are sure the uterus is empty." . . . "A little pelvic search will usually discover the whereabouts of the fetus," he speaks, of course, of a viable fetus. If the fetus is dead, even operation may discover it only accidentally, after most thorough irrigation.

The diagnosis of extra-uterine pregnancy after full period of gestation and fatal death is considered at length. Here the author relies for his authority upon Parry and Tait. The former he quotes freely, and uses all the salient features of the latter's description, with scarcely sufficient acknowledgment. These are briefly : hemorrhage at false labor, and diminution in size afterwards, together with a tumor always intimately associated with the uterus. These symptoms are constant. The diagnosis of suppuration in the fetal sac need not be here considered. Dr. Strahan follows Tait in all the essential features. His citation of cases is interesting, and well illustrates the new pathology of the condition, as demonstrated by Hart and Carter. To those who have not noticed the case of Gervis previously, (*British Medical Journal*, p. 818, Oct. 30, 1886,) we would urge its perusal, as once more forcibly illustrating the uncertainty of exact diagnosis. The differential diagnosis of extra-uterine pregnancy from other abdominal and pelvic growths *enumerates the following conditions likely to lead to error in extra-uterine pregnancy: normal pregnancy with retroversion or retroflexion, pregnancy in one case of a bifid uterus, ovarian tumor, cyst of broad ligament,* Fallopian distention, uterine tumors, pelvic hematocele (simple), pelvic inflammatory exudations or abscesses (simple), cancer of the pelvis or peritoneum—ten in all.

For the consideration of these, we must refer the reader to the essay. Time does not permit their notice in detail. To the list the author should have added, miscarriage in normal pregnancy, complicated with accident or antecedent pelvic inflammation. He errs also in giving pain with fever as a differential sign of pyosalpinx. These are not uniformly present. One or the other may be present alone. Pain is oftener more uniform than fever. Dr. Strahan's advice, (p. 63,) that when in doubt wait at least until the possibility of normal pregnancy can be eliminated, and then have the tumor removed, will if followed prevent errors, which, when they occur, are regarded as a reproach to surgery. The differential diagnosis of cancer from ectopic gestation is interesting; how easily two pathological processes, utterly antipodal in their nature, can perplex, and defeat the most skilful diagnostician in his efforts to differentiate them!

Part II. of the essay deals with the treatment of extra-uterine pregnancy in its various stages. The treatment of early extra-uterine pregnancy when rupture occurs, is that of Mr. Tait, backed up by Tait's arguments. Operation is the treatment.

Of the plan to be pursued, previous to rupture if diagnosis be made, (p. 74,) at once leads to a discussion of the electrical treatment versus surgical interference. Of the methods, such as electrolytic puncture, injection of poisons into the fetal sac, and simple tapping, we shall pass without notice, as unworthy at the present time of aught but the severest condemnation, no matter by whom proposed, or by whom attempted.

Though an authority so well known as Barnes advocates the method of puncture, the records of the procedure are so ghastly that he should, like the Scriptural maniacs who dwelt among the sepulchres, be permitted to enjoy its horrors alone.

The removal of the fetus through the vagina, has been conclusively shown by Herman to be inadvisable. If operation is to be done, the abdominal section is to be preferred. (Tait, "Lectures," p. 84, quoted also by Strahan, p. 122.) So far as the electrical treatment is concerned, its opponents and advocates are equally positive—the first as to its faults, dangers, and defects; the second as to its safety, certainty, and ease of application. Unfortunately, it is proven past dispute that the current does not always kill the fetus, just as it is demonstrated, that it has killed fetuses which did not exist, as Wylie's well-known case attests. It has been used in normal pregnancy to kill an ectopic fetus, by a physician whose previous diagnosis and cure by the electric current of an ectopic pregnancy has been reported and dwelt upon complacently by the supporters of this treatment. There have been ruptures after its use, and suppurating cysts. Moreover, we have shown that what is claimed as positive diagnosis by the electricians, is practically no diagnosis at all—witness the cases of Thomas. But if the diagnosis is correct, what can be said of a treatment that allows a dangerous mass to remain within the abdomen?

The absolute removal of an ectopic cyst previous to rupture, is as simple as the simplest abdominal operation. The advocates of this treatment dwell upon the dangers of abdom inal operations, while they relegate only the dangerous cases of rupture to operation, and claim wonders for their treatment, where, if the condition actually exists for which they apply it, surgical interference would be absolutely safe, as absolutely sure, with no danger of subsequent complications. Early or late, be the child living or dead, we submit that our preference is for positive surgical interference, without one moment of unnecessary delay. The references to this portion of the subject are well collated, and give a remarkably clear exposition of the argument. The details of all the operative requirements are sufficiently well known by all who have seen Tait's lectures, without repeating them here. The question of the after-growth of the placenta, as well as some other points of interest did space permit, must be passed without mention.

As a whole, Dr. Strahan has produced a valuable essay, so far as collecting a series of cases illustrative of the subject is concerned. From the abundance of material at his hand, he might have produced a much more telling statement of the subject. His acceptance of some cases as proving what they claim, is at times too ready. His repetitions tend to weaken what otherwise would be a strong statement of his argument. The grammar, not to be hypercritical, is often faulty, while throughout there is a tendency to a mode of expression distasteful in a scientific production.

The whole argument, except the acceptance of the possibility of diagnosis of ectopic pregnancy previous to rupture, is the opinion of Mr. Tait, reinforced by an extensive bibliography. It is nevertheless valuable because collating many facts, and creditable because exhibiting industrious research.

---

Tarrant's Seltzer Aperient has become such a standard that its qualities are well understood by most physicians. It is particularly useful in lithemic and gouty conditions, and is altogether one of the most agreeable alkaline and laxative preparations that we know of. It is a most excellent substitute for the various fashionable mineral waters that are so commonly prescribed, and is even a more efficient remedy than most of them.

